# Anti-angiogenic effect of the total flavonoids in *Scutellaria barbata* D. Don

**DOI:** 10.1186/1472-6882-13-150

**Published:** 2013-07-01

**Authors:** Zhi-Jun Dai, Wang-Feng Lu, Jie Gao, Hua-Feng Kang, Yu-Guang Ma, Shu-Qun Zhang, Yan Diao, Shuai Lin, Xi-Jing Wang, Wen-Ying Wu

**Affiliations:** 1 Department of Oncology, the Second Affiliated Hospital of Xi’an Jiaotong University, Xi’an 710004, China; 2 Department of Surgical Oncology, Shangluo Central Hospital, Shangluo 726000, China; 3 Department of Nephrology, the Second Affiliated Hospital of Xi’an Jiaotong University, 710004, Xi’an, China; 4 Department of Pharmacology, the Second Affiliated Hospital of Xi’an Jiaotong University, Xi’an 710004, China

**Keywords:** *Scutellaria Barbata*, Angiogenesis, Hepatocellular Carcinoma, Human Umbilical Vein Endothelial Cells

## Abstract

**Background:**

Angiogenesis is closely related to the growth, invasion and metastasis of tumors, also considered as the key target of anticancer therapy. *Scutellaria barbata* D. Don (*S. barbata*), a traditional Chinese medicine, is being used to treat various diseases, including cancer. However, the antitumor molecular mechanism of *S. barbata* was still unclear. This study aimed to investigate the inhibitory effects of the total flavones in *S. barbata* (TF-SB) on angiogenesis.

**Methods:**

Human umbilical vein endothelial cells (HUVECs) were treated with various concentrations of TF-SB. Cell viability was examined using the MTT assay. The scratch assay was used to detect the migration of HUVECs after treatment with TF-SB. The ability of HUVECs to form network structures *in vitro* was demonstrated using the tube formation assay. The chick embryo chorioallantoic membrane assay was performed to detect the *in vivo* anti-angiogenic effect. The expression of VEGF was measured by the enzyme-linked immunosorbent.

**Results:**

Results showed that TF-SB inhibited the proliferation and migration of HUVECs in a dose- dependent manner. Simultaneously, TF-SB significantly suppressed HUVEC angiogenesis *in vitro* and *in vivo*. Furthermore, VEGF was downregulated in both HUVECs and MHCC97-H cells after TF-SB treatment.

**Conclusion:**

TF-SB could suppress the process of angiogenesis *in vitro* and *in vivo*. TF-SB potentially suppresses angiogenesis in HUVECs by regulating VEGF. These findings suggested that TF-SB may serve as a potent anti-angiogenic agent.

## Background

The incidence of hepatocellular carcinoma (HCC) in worldwide is on the rise. According to global statistics, approximately 7 million of people die each year from liver cancer which includes HCC [1]. The selection of HCC treatment depends on the tumor heterogeneity, biological behavior, and liver function [2]. Chemotherapy is one of the main methods for the treatment of HCC because it can completely destroy cancer cells. However, chemotherapy may cause severe simultaneous side-effects and often lead to multidrug resistance [3]. Therefore, novel anti-cancer pharmaceutical products must be developed; these products may be obtained from Chinese herbal medicines [4,5]. Several herbs have been found to have anti-tumor activity and become the main sources of anti-cancer drugs [6].

Angiogenesis is the formation of new blood vessels from existing vasculature. This process has been associated with the growth and dissemination of solid tumors [7]. The complex process of tumor angiogenesis is associated with basal membrane injury, endothelial cell proliferation, cell migration, and the activation of angiogenic factors, among others [8]. The expressions of several cytokines, such the vascular endothelial growth factor (VEGF) and angiopoietin (Ang), is involved in this formation process [9-11]. Cytokines are important regulators of angiogenesis [12]. Therefore, natural herbs have been considered as potential novel sources of compounds to inhibit vascular [13].

*Scutellaria barbata* D. Don (*S. barbata*) is a herb used in traditional Chinese medicine, which is widely distributed in certain areas of China and Korea. *S. barbata* has known anti-inflammatory and anti-tumor effects. Thus, this herb has been clinically used for treating inflammatory diseases and cancer. The crude extracts of *S. barbata* have demonstrated inhibitory effects on numerous human cancers *in vitro,* including hepatoma, colon cancer, lung cancer, and breast cancer [14-18]. Our previous study confirmed that the extract of *S. barbata* is a potent inhibitor in hepatoma *in vitro* and *in vivo*[19]. To date, several flavonoids, alkaloids, polysaccharides, and steroids from *S. barbata* have been characterized [20-24]. In the present study, we investigated the anti-angiogenic effects of total flavonoids of *S. barbata* (TF-SB) in human umbilical vein endothelial cells (HUVECs) and the human HCC cell line MHCC97-H.

## Methods

### Reagents

Fetal bovine serum (Gibco BRL, Rockville, MD, USA); DMEM medium (Gibco, USA); 3-(4,5-Dimethylthiazol-2-yl)-2,5-diphenyl tetrazolium bromide (MTT) was purchased from Sigma-Aldrich (St. Louis, MO,USA); Matrigel (BD Biosciences, San Jose, CA, USA); human VEGF ELISA kit was purchased from WuHan Boshide Biotechnology Co, Ltd. (WuHan, China).

### Preparation of TF-SB from *Scutellaria barbata* D. Don

Dried plant materials of *S. barbata* were purchased from Yi Shan Tang Chinese Herbal medicine store (Xi’an, China) and authenticated according to the descriptions found in the Chinese Pharmacopoeia. The original herb was identified as *Scutellaria barbata* D. Don (SB) by Run-Xia Liu at Medical School of Xi’an Jiaotong University (Xi’an, China). The voucher samples, ZLK-ZY-05 (*S. barbata*) was deposited at the department of oncology, the Second Affiliated Hospital of Xi’an Jiaotong University.

The material was coarsely ground before extraction. A total of 300 g of the material was extracted twice with 95% ethanol for 3 h in 50°C. The infusion was filtered through a 1-mm pore-size filter. The leftover on the filter was collected after evaporated. The crude extract was isolated by AB-8 macroporous adsorption resin column in which 70% aqueous ethanol was used to elute flavonoids. After treatment with AB-8 resin, the flavonoids purity increased with a recovery of 69%. The total flavonoids were stored at 4°C for use.

### Cell line and cell culture

MHCC97-H cells and HUVECs were purchased from the Liver Cancer Institute of Fudan University (Shanghai, China). The cells were grown in DMEM maximal medium containing 10% inactived fetal bovine serum. Both cell lines were cultured at 37°C in 5% CO_2_ under humidified environment.

### MTT assay for the cell viability of HUVEC cells

Viability of HUVECs was assessed by the MTT assay. Cells were seeded into 96-well plates at the density of 1 × 10^4^ cells/well. After 12 h, the cells were treated with TF-SB in different concentrations (0, 20, 40, 80 and 160 μg/mL) for 48 h or 72 h, respectively. MTT were applied to each well after treatment. The supernatant were removed after 4 h incubation. Subsequently, DMSO were added to each. The supernatants were removed carefully and 150 μL of dimethyl sulfoxide (DMSO) were added to each well. The absorbance was measured at 490 nm through an Enzyme-labeling instrument (ELX800, Bio-Tek, Winooski, VT, USA). This assay was performed in triplicate. The results represented the average value of aborbance from three independent experiments done over multiple days.

### *In vitro* scratch assay

We used the *in vitro* scratch assay to assess the activity of TF-SB on migration of HUVECs [25]. HUVECs were seeded in 12-well plates (2 × 10^5^/well) with complete medium overnight to obtain a full confluent monolayer. After 24 h, the cells were scraped away vertically 24 h later by pipette tip. Each well was washed twice with PBS to remove debris, and then further incubated for 24 h in serum-free DMEM medium with different concentrations of TF-SB (0, 40, 80 and 120 μg/mL). The distances between the 2 edges of the scratch were photographed on each well using inverted microscope at a magnification of 100× and analyzed quantitatively.

### Tube formation assay

The ability of HUVECs forming network structures was tested by tube formation assay. As previously described [26], 96-well plates were plated with 50 μL matrigel and allowed to polymerize at 37°C for 30 min. HUVECs were subsequently seeded on the matrigel followed by addition of different concentrations of TF-SB (0, 40, 80 and 120 μg/mL) and incubation for 9 h at 37°C. The tube-like structures were photographed on each well using a phase-contrast microscope (Olympus, Tokyo, Japan) at a magnification of 100 ×. To quantify the results, we counted the number of branch points, in which at least 3 tubes joined.

### Chick chorioallantoic membrane (CAM) assay

The CAM assay was performed as previously described [25]. 60 fertilized chicken eggs (14 ± 2 d) were purchased from Huxian chicken farm (Xi’an, China). The animals were housed and handled in strict accordance with the guidelines of the institutional and national Committees of Animal Use and Protection. The protocol was approved by the Committee on the Ethics of Animal Experiments of Xi’an Jiaotong University College of Medicine (Certificate No. 22–9601018). All efforts were made to minimize animals’ suffering and to keep the numbers of animals used to a minimum.

Briefly, the eggs were incubated at 37°C in 40–60% humidity for 96 h. And then, the eggs were randomly divided into four groups that were treated with different concentrations of TF-SB. After 7 days, a window (1 × 1.5 cm^2^) was opened in the shell to expose a part of the CAM. Different concentrations of TF-SB samples in 20 μL PBS was loaded onto sterilized gelatin sponges (2 mm^2^) that was then applied to the CAM. After 48 h of incubation, the neovascular numbers in the CAM around the sponges were photographed with an anatomical microscope (YZ20T4 type). The CAM of the sponge around were observed after hematoxylin and eosin (HE) staining [26]. The relationship between leukocyte infiltration and angiogenesis were analyzed quantitatively.

### Measurement of VEGF levels by ELISA

We used ELISA assay to measure the variation of VEGF levels in MHCC97H cells and HUVECs. The supernatant was collected from different treatment groups. The VEGF level in MHCC97-H cells and HUVECs were measured by ELISA kit (Boshide) according to the manufacturer’s instructions. The each well was plated with 0.1 mL diluted samples in samples buffer and incubated 90 min at 37°C. Next, 100 μL anti-human VEGF antibody was added and incubated for another 60 min. After washing with PBS for three times, 90 μL TMB color liquid was added in the dark for 30 min. And then, the absorbance was measured at 450 nm after TMB Stop Solution was applied. All measurements were performed for three times. The data represented average of absorbance value from three independent experiments.

### Statistical analysis

Data were presented as Mean ± standard deviation (SD). Statistical analysis of the data were performed with Student’s *t*-test,one-way analysis of variance (ANOVA) test and linear regression analysis using the Statistical Package for Social Sciences version 13.0 (SPSS Inc, Chicago, IL). *p* value < 0.05 was considered statistically significant.

## Results

### Identification of TF-SB by high performance liquid chromatography (HPLC)

**Figure 1:**
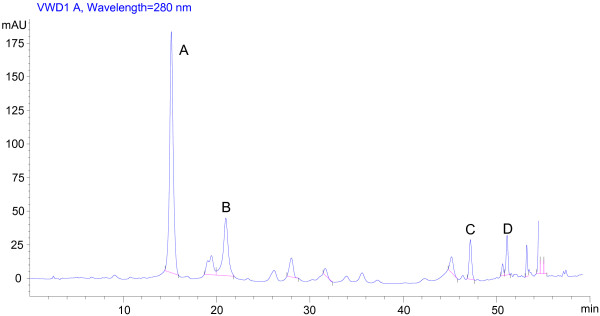
**HPLC analysis of TF-SB.** There was a main peak in HPLC, which was identified as scutellarin (**A**). There were also some other flavonoids in TF-SB which were identified as apigenin (**B**), baicalein (**C**), luteolin (**D**). (Wavelength = 280 nm).

The components of TF-SB were identified using HPLC. As shown in Figure 1, the main peak was identified as scutellarin (A). Other identified flavonoids in TF-SB included apigenin (B), baicalein (C), luteolin (D). The contents of flavonoids A-D were 67.2%, 8.7%, 4.6% and 4.3%, respectively.

### Effects of TF-SB on the cell viability of HUVECs

**Figure 2:**
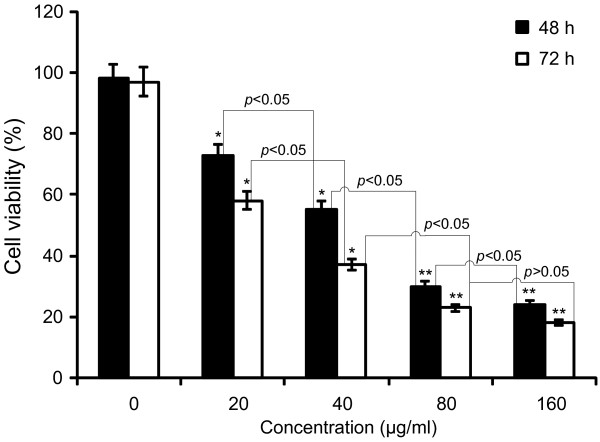
**Growth inhibiting effects of TF-SB on HUVECs.** Cell viability was determined by MTT method and treated with different concentrations drug for 48 or 72 h. This assay was performed in triplicate (*p* < 0.05, ANOVA analysis). ^*^p < 0.05, ^**^p < 0.01 versus the control group.

The MTT assays showed that the cell viabilities of the TF-SB treated groups (20, 40, 80 and 160 μg/mL) were suppressed by 24.3% ± 0.1%, 30.9% ± 1.5%, 55.4% ± 0.9% and 73.2% ± 0.6% respectively, after 48 h treatment (Figure 2). The inhibition rate in the TF-SB treated groups was further decreased after treatment for 72 h, while the inhibitory rate of the 160 μg/mL group reached 78.1% ± 0.6%. Therefore, the anti-proliferative effects of TF-SB on HUVECs occured in a time- and dose-dependent manner.

### Effect of TF-SB on the migration of HUVECs

**Figure 3:**
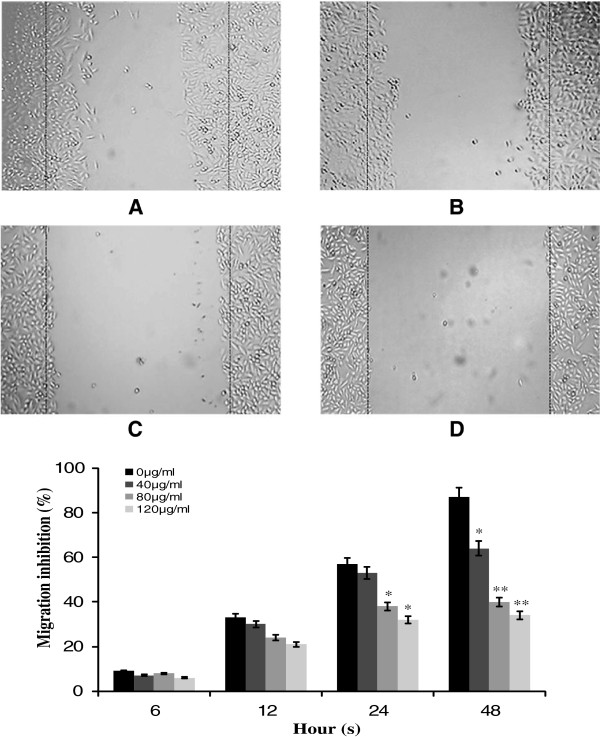
**Effects of TF-SB on the cell migration of HUVECs.** Cell migration was analyzed by the scratch assay. HUVECs were treated with various concentrations of TF-SB (40, 80 and 120 μg/mL) for 48 h. **A**: blank control group; **B**: 40 μg/mL TF-SB group; **C**: 80 μg/mL TF-SB group; **D**: 120 μg/mL TF-SB group. The images were captured under a phase-contrast microscope at a magnification of 100×. Values represent mean ± SD from three independent experiments. ^*^p < 0.05, ^**^p < 0.01 versus the control group.

Endothelial cell migration is a necessary step of angiogenesis [9]. In the present study, the effects of TF-SB on HUVECs migration were determined using the scratch assay. As shown in Figure 3, cell migration in TF-SB groups was inhibited in various degrees after 48 h of treatment. The maximum inhibition was achieved by the group that received 120 μg/mL TF-SB, which was higher than control group (*p* < 0.05) or those with 40 μg/mL treatment (*p* < 0.05). The cell migration of HUVECs was inhibited in a dose-dependent manner by treatment with TF-SB for 48 h.

### TF-SB inhibits HUVEC neovascularization

**Figure 4:**
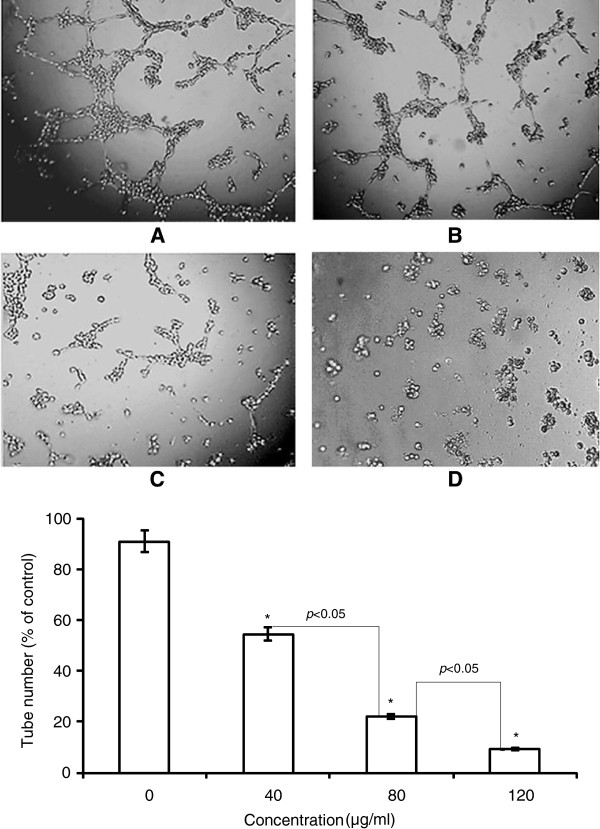
**The effect of TF-SB on HUVEC tube formation.** HUVECs were seeded on Matrigel-coated 96-well plates and incubated in the diluted medium containing different concentrations of TF-SB for 9 h at 37°C. **A**: blank control group; **B**: 40 μg/mL TF-SB group; **C**: 80 μg/mL TF-SB group; **D**: 120 μg/mL TF-SB group. The images were captured under a phase-contrast microscope at a magnification of 100 × and observed the network-like structures. Values represent mean ± SD from three independent experiments. ^*^p < 0.05, ^**^p < 0.01 compared with the control group.

The formation of tube-like structures is an essential step in angiogenesis. This process involves matrix degradation, rearrangement and apoptosis of endothelial cells [27]. Therefore, HUVECs angiogenesis was observed using the tube formation assay. As shown in Figure 4, the capillary tube structures were observed in the basal membrane of the control group after HUVECs were placed in the wells. By contrast, the TF-SB treatment significantly reduced the formation of tube-like structures in a dose-dependent manner. Only a few tube-like structures were formed in the 120 μg/mL TF-SB treated group.

### Effect of TF-SB on angiogenesis *in vivo*

The chicken chorioallantoic membrane (CAM) assay has been widely used for developmental and post-developmental studies of angiogenesis because of the easy access to the vascularized CAM [28,29]. Moreover, animal experimentation licenses are not compulsory for chicken embryo experiments in many countries. Furthermore, the CAM assay is rapid, inexpensive, and suitable for the large-scale screening of potential angiogenesis regulators [30].

**Figure 5:**
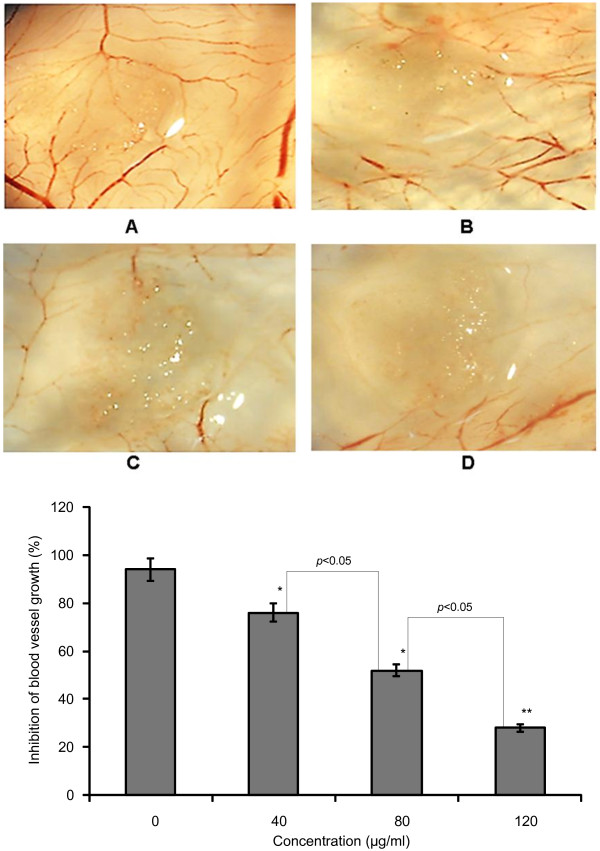
**The effects of TF-SB on the angiogenesis of CAM.** The chick chorioallantoic membrane of 7-day-old chick embryos were treated with various concentrations of TF-SB and incubated for 48 h. **A**: blank control group; **B**: 40 μg/mL TF-SB group; **C**: 80 μg/mL TF-SB group; **D**: 120 μg/mL TF-SB group. The angiogenesis around the gelatin sponges was photographed with an anatomical microscope. Values represent mean ± SD from fifteen eggs. ^*^p < 0.05, ^**^p < 0.01 compared with the control group.

In this study, we used a CAM model to confirm the effect of TF-SB on angiogenesis *in vivo*. After treatment for 48 h, the blood vessel structures were observed using an anatomical microscope. As shown in Figure 5, a normal vascular pattern with numerous branching was observed in the control group. The total number of blood vessels in the TF-SB treatment was significantly decreased, as compared with the control group (*p* < 0.05). The results indicated that TF-SB could suppress angiogenesis *in vivo*.

### Relationship between the leukocyte infiltration count and blood vessel formation

**Figure 6:**
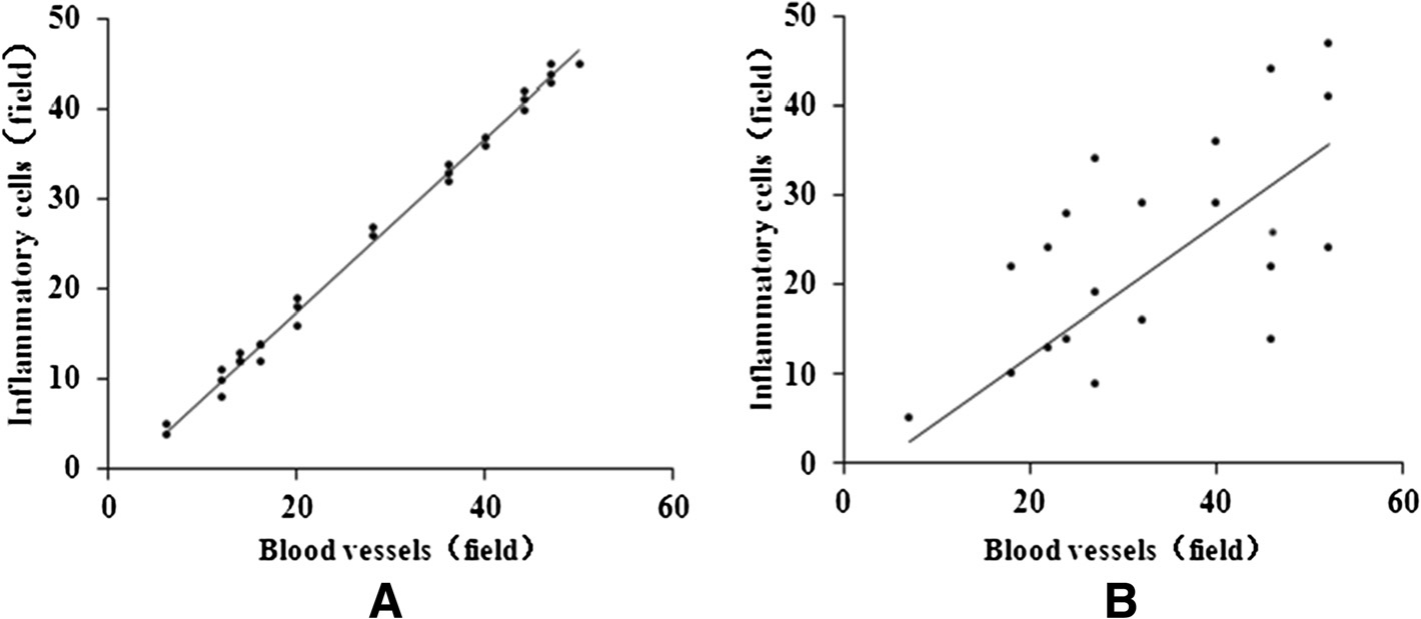
**The relationship between leukocyte infiltration count and formation of blood vessels.** The leukocyte infiltration and blood vessels on the CAM were observed under the microscope by HE staining. and no correlation between the formations of big blood vessels. **A**: The leukocyte infiltration was positively correlated with small blood vessels (*r* = 0.883, *p* < 0.05); **B**: The leukocyte infiltration had no correlation with formation of big blood vessels (*r* = 0.067, *p* > 0.05).

The infiltration of macrophages, lymphocytes, and mast cells often presented in tumors microenvironment because these cells may contribute to tumor progression. Previous studies have suggested that these inflammatory cells promote the neoplastic progression by stimulating tumor revascularization and other processes related to tumor angiogenesis [31]. In the present study, the leukocyte infiltration count for the surrounding sponge angiogenesis was observed on the CAM by hematoxylin and eosin (HE) staining. As shown in Figure 6, leukocyte infiltration was not correlated with the formation of large blood vessels. However, the infiltration was positively correlated with the formation of small blood vessels.

### TF-SB suppresses the expression of VEGF in both MHCC97-H cells and HUVECs

**Figure 7:**
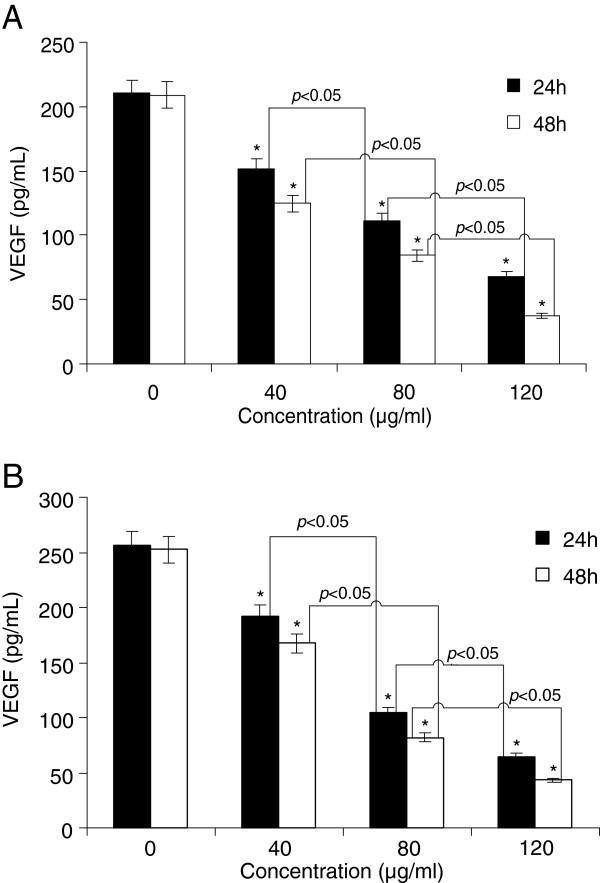
**Effect of TF-SB on the expression of VEGF in both MHCC97-H cells and HUVECs.** Cells were treated with different concentrations of TF-SB for 24 h or 48 h. The protein secretion levels of VEGF were examined by ELISA in MHCC97-H cells (**A**) and HUVECs (**B**). The VEGF expression in the two cell lines were significantly reduced. This assay was performed in triplicate. *p < 0.05 versus the control group.

VEGF is a potent mitogen responsible for the induction of angiogenesis [32]. The humanized monoclonal antibody of VEGF, named bevacizumab, has been used to treating several cancers [33]. In this study, variations of the VEGF levels were measured using ELISA. The cells were treated with different concentrations of TF-SB for 24 or 48 h. The VEGF was then detected in the supernatant culture media. As shown in Figure 7, VEGF expression levels were clearlyly decreased after TF-SB treatment in MHCC97-H cells and HUVECs. Furthermore, the results showed that the VEGF expression levels were correlated with the various concentrations of TF-SB (*p* < 0.05).

## Discussion

Novel treatments, such as targeted therapy and gene therapy have become an important means of comprehensive cancer treatment for various tumors. However, these therapies have not been widely used because of their limited efficiency and high cost. Traditional Chinese medicine has been used in anti-tumor treatment for thousands of years. These medicines have been used to enhance immune function, and reduce side effects, as well as to prevent recurrence and metastasis for cancer patients. The anticancer mechanism and activity of extracts from Chinese herbs have been reported *in vitro*. For example, BZL101 is an aqueous extract of *S. barbata* with anticancer properties against several human cancers [34]. The crude extract of *S. barbata* similarly has anticancer and anti-angiogenic activity *in vitro* and *in vivo*[19,35].

*S. barbata* is one of the conventional anticancer drugs in China because of its significant anti-tumor activity and inhibition of angiogenesis [36]. *S. barbata* is effective against a wide range of tumors. The extracts of *S. barbata* greatly could greatly inhibit the cell growth in lung cancer, leukemia, colon cancer, hepatoma, and skin cancer [15-18]. Lee *et al.* reported that *S. barbata* decreased the proliferation of myometrial and leiomyomal cells originally promoted by HCG [37]. The chemical composition of *S. barbata* includes flavonoids, diterpenoids, and polysaccharides. Flavonoids are considered the main anti-tumor component of *S. barbata*. In our study, we found that TF-SB could inhibit angiogenesis based on the proliferation, migration and tube formation of endothelial cells. Our results showed that TF-SB inhibited the proliferation and migration of HUVECs in a dose- dependent manner. Thus, TF-SB could significantly suppress the progress of angiogenesis in HUVECs.

Tumor neovascularization is defined as the process of new blood vessel formation in solid neoplasms [27]. The activation of angiogenic pathways is required for tumor spreading, and the proliferation of metastatic cells in distant organs [38]. In a phase IB, multicenter clinical trial of the drug Bezielle in the USA, an aqueous extract of *S. barbata*, was safely administered to the patients and demonstrated promising clinical evidence of anticancer activity in a heavily pretreated population of women with metastatic breast cancer [39]. The ethanol extract of *S. barbata* could inhibit tumor angiogenesis in a colorectal cancer mouse xenograft model by suppressing the SHH pathway [40]. In the present study, angiogenesis of HUVECs was observed through tube formation assay. We found that TF-SB could significantly suppress the process of angiogenesis of HUVECs.

Inflammation, cytokine activation, and angiogenesis are common features of the tumor microenvironment during the progression of malignancy [41]. These biological processes often share mutual pathways related to cancer progression [42]. Inflammatory cells are macrophages or monocytes induced by various cytokines to become tumor-associated macrophages (TAM) during tumorigenesis. These cells are considered contributing factors for creating the required microenvironment for angiogenesis [43]. In the present study, we found that TF-SB inhibited the angiogenesis of CAM *in vivo*. Simultaneously, the surrounding sponge layer had numerous infiltrating inflammatory cells by HE staining. A positive correlation was found between leukocyte infiltration and the abundance of small blood vessels.

VEGF may be combined with specific endothelial cell receptors, which are secreted by the autocrine and paracrine pathways to promote angiogenesis [44]. In the present study, the results showed that TF-SB downregulated the expression of VEGF in the MHCC97-H cells and HUVECs.

## Conclusion

In conclusion, TF-SB treatment could significantly suppress the process of angiogenesis in HUVECs grown on matrigels *in vitro*, and the angiogenesis of CAM *in vivo*. However, TF-SB is composed of several compounds, including scutellarin, apigenin, baicalein and luteolin, among others. The anticancer effects of these individual components or of various combined ingredients remain unknown. Further experimental studies are required to clarify the anticancer molecular mechanisms of TF-SB treatment.

## Abbreviations

HCC: Hepatocellular carcinoma; VEGF: Vascular endothelial growth factor; HUVECs: Human umbilical vein endothelial cells; MTT: 3-(4,5-Dimethylthiazol-2-yl)-2,5-diphenyl tetrazolium bromide; CAM: Chick embryo chorioallantoic membrane; TF-SB: Total flavones of Scutellaria barbatae; HPLC: High performance liquid chromatography.

## Competing interests

The authors declare that they have no competing interests.

## Authors’ contributions

DZJ, WXJ and WWY designed the research. DZJ, LWF, GJ, KHF and MYG performed the experiments throughout this research. ZSQ, DY and LS contributed to the reagents, and participated in its design and coordination. DZJ and GJ analyzed the data; DZJ and LWF contributed to the writing of the manuscript. Co-first authors: DZJ, LWF and GJ. All authors have read and approved the final manuscript.

## Pre-publication history

The pre-publication history for this paper can be accessed here:

http://www.biomedcentral.com/1472-6882/13/150/prepub
